# Evolution of Graves’ Disease during Immune Reconstitution following Nonmyeloablative Haploidentical Peripheral Blood Stem Cell Transplantation in a Boy Carrying Germline *SAMD9L* and *FLT3* Variants

**DOI:** 10.3390/ijms23169494

**Published:** 2022-08-22

**Authors:** Peng Peng Ip, Li-Hua Fang, Yi-Ling Shen, Kuan-Chiun Tung, Ming-Tsong Lai, Li-Ying Juan, Liuh-Yow Chen, Rong-Long Chen

**Affiliations:** 1 Institute of Molecular Biology, Academia Sinica, Taipei City 115, Taiwan; 2 Department of Pharmacy, Koo Foundation Sun Yat-Sen Cancer Center, Taipei City 112, Taiwan; 3 Taiwan Genome Industry Alliance Inc., Taipei City 115, Taiwan; 4 Division of Endocrinology, Department of Internal Medicine, Koo Foundation Sun Yat-Sen Cancer Center, Taipei City 112, Taiwan; 5 Department of Pediatric Hematology and Oncology, Koo Foundation Sun Yat-Sen Cancer Center, Taipei City 112, Taiwan

**Keywords:** *FLT3* variant, Graves’ disease, HLA-B*46:01, HLA-DRB1*09:01, nonmyeloablative haploidentical peripheral blood stem cell transplantation, *SAMD9L* variant, severe aplastic anemia

## Abstract

Graves’ disease, characterized by hyperthyroidism resulting from loss of immune tolerance to thyroid autoantigens, may be attributable to both genetic and environmental factors. Allogeneic hematopoietic stem cell transplantation (HSCT) represents a means to induce immunotolerance via an artificial immune environment. We present a male patient with severe aplastic anemia arising from a germline *SAMD9L* missense mutation who successfully underwent HSCT from his HLA-haploidentical *SAMD9L* non-mutated father together with nonmyeloablative conditioning and post-transplant cyclophosphamide at 8 years of age. He did not suffer graft-versus-host disease, but Graves’ disease evolved 10 months post-transplant when cyclosporine was discontinued for one month. Reconstitution of peripheral lymphocyte subsets was found to be transiently downregulated shortly after Graves’ disease onset but recovered upon antithyroid treatment. Our investigation revealed the presence of genetic factors associated with Graves’ disease, including HLA-B*46:01 and HLA-DRB1*09:01 haplotypes carried by the asymptomatic donor and germline *FLT3* c.2500C>T mutation carried by both the patient and the donor. Given his current euthyroid state with normal hematopoiesis, the patient has returned to normal school life. This rare event of Graves’ disease in a young boy arising from special HSCT circumstances indicates that both the genetic background and the HSCT environment can prompt the evolution of Graves’ disease.

## 1. Introduction

Autoimmune Graves’ disease, featuring a failure to tolerate thyroid-derived antigens, is characterized by hyperthyroidism and biochemically elevated thyroxine or suppressed thyrotropin together with the presence of thyroid-stimulating thyrotropin receptor antibodies [1,2]. Genetic predisposition and nongenetic factors have been linked to Graves’ disease, resulting in overproduction of thyroid hormones by thyroid epithelial cells due to overactive thyrotropin receptor. Together with drastically amplified intrathyroidal cytokine production by infiltrating immune cells, thyrotropin receptor overactivity further activates and sustains inflammation to alter the behavior of thyroid epithelial cells [2,3,4]. Allogeneic hematopoietic stem cell transplantation (HSCT) procedures, including cytoreductive therapies to condition and modulate graft–host interactions, represent artificial platforms for immune reconstitution and tolerance induction. However, significantly, chronic graft-versus-host disease (GVHD) may develop if immune tolerance induction is aberrant [5]. Several reports have described Graves’ disease as having evolved following allogeneic HSCT for aplastic anemia, and they proposed associated factors for the rare coincidence including GVHD, use of rabbit antithymocyte globulins (rATG), and adoptive transfer of donor pathogenic lymphocytes [6,7,8,9,10,11]. Here, we report a case of coincident Graves’ disease during post-transplant immune reconstitution in a boy displaying bone marrow failure and carrying germline *SAMD9L* and *FLT3* variants. This patient had undergone father-to-son haploidentical peripheral blood stem cell (PBSC) transplantation with nonmyeloablative conditioning and high-dose post-transplant cyclophosphamide (PTCy) treatment.

## 2. Case Report

**Figure 1:**
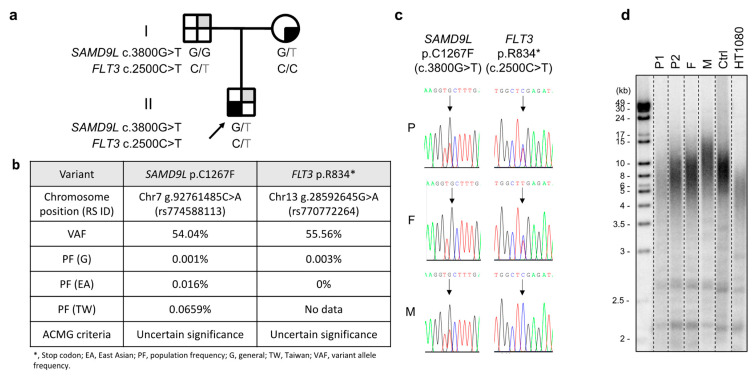
(**a**) Pedigree of the affected family. Gray-shaded quarters represent carriers of the *FLT3* c.2500C>T mutation, and black-shaded quarters represent carriers of the *SAMD9L* c.3800G>T mutation. Squares and circle represent male and female subjects, respectively. (**b**) Characteristics of the variants. American College of Medical Genetics and Genomics (ACMG) criteria for variant interpretation encompass a five-tier classification: Pathogenic, Likely pathogenic, Uncertain significance, Likely benign, and Benign. (**c**) Sanger DNA sequencing of *SAMD9L* exon 5 on chromosome 7 and *FLT3* exon 20 on chromosome 13 from peripheral blood cells taken from subjects of the studied pedigree. Patient (P), patient’s father (F), patient’s mother (M). (**d**) Telomere length analysis (using terminal restriction fragment assay) of DNA from leukocytes of the patient pre-transplant (P1) and 1.5 years post-transplant (P2) as well as from the patient’s father (F), patient’s mother (M), a healthy control (Ctrl), and from HT1080 fibrosarcoma cancer cells. The patient pre-transplant (P1) possessed shorter and heterogeneous telomeres compared to the control (Ctrl). However, the telomeres of the patient 1.5 years post-transplant (P2) became more normal and similar to those of his father (F). For illustrative purposes, the blot has been cropped (original provided in the Supplementary Materials; Figure S1).

The patient, an eight-year-old boy and the sole child of nonconsanguineous parents with Chinese Han ethnicity, was diagnosed with severe aplastic anemia in October 2019. Treatments were initiated soon after diagnosis, including intravenous immunoglobulin (1 g per kg; 2 doses in October 2019), eltrombopag (up to 75 mg/day administered from October 2019 to April 2020), rATG (thymoglobulin 2.5 mg/kg/day for 5 days in October 2019), cyclosporine (administered by titrating the serum level between 200 and 300 ng/mL from October 2019), and intermittent granulocyte-colony-stimulating factors. The hematologic response was deemed inadequate, with a transfusion dependency requiring red blood cells to be provided monthly and platelets biweekly. Persistently low neutrophil counts (ranging between 0.35 and 0.75 × 10^9^/L) were also observed despite bone marrow cellularity increasing from <1% at diagnosis to 5–10% (July 2020). No physical anomalies or laboratory evidence of cytogenetic changes or Fanconi anemia were detected in July 2020 upon transfer to Koo Foundation Sun Yat-Sen Cancer Center, Taipei, although morphological myelodysplasia in the bone marrow was a concern (data not shown). Whole-exome sequencing revealed a germline *SAMD9L* c.3800G>T; p.(Cys1267Phe) mutation with a very low population allele frequency, which was validated by Sanger sequencing (Figure 1a–c). The patient’s bone marrow failure syndrome was assumed to be linked to this mutation. Both parents displayed normal hematopoiesis despite the patient’s mother also carrying the same *SAMD9L* mutation. Telomere length was also found to be short in the patient relative to an age-matched control (Figure 1d).

As HLA-matched donors were unavailable, a decision was made to pursue haploidentical PBSC transplantation into the patient from his father, who lacked the *SAMD9L* mutation, as salvage therapy. The conditioning treatments consisted of intravenous (IV) fludarabine (30 mg/m^2^ daily from day −6 to −2) and cyclophosphamide (14.5 mg/kg daily IV from day −6 to −5), as well as 4 Gy total body irradiation on day −1. In August 2020, the boy received PBSCs comprising 14.5 × 10^8^ total nucleated cells/kg, 14.0 × 10^6^ CD34^+^ cells/kg and 1.4 × 10^8^ CD3^+^ cells/kg. The donor and patient were HLA-haploidentical (A 11/-, B 46/51, C 01/15, DQ 03/-, DR 09/11 to A 0201/1101, B 3901/5102, Cw 0702/1502, DQB1 0301/-, DRB1 1101/-) and ABO-nonidentical (O to B). For GVHD prophylaxis, the patient received PTCy (cyclophosphamide 50 mg/kg daily IV on days +3 and +4), cyclosporine (for 9 months by adjusting the serum level at 200–300 ng/mL during first month post-transplant and at 150–250 ng/mL thereafter), and mycophenolate mofetil (500 mg orally twice daily until day +30) from day +5. Ursodiol (until day +100) was given to prevent sinusoidal obstruction syndrome, and baktar (for 9 months), valacyclovir (until day +30), entecavir (for 1 year), and letermovir (until day +100) were provided to prevent post-transplant pneumocystis infection or reactivations of herpes simplex, hepatitis B, and cytomegalovirus, respectively. Neutrophils engrafted on day +14, with 100% donor chimerism thereafter. No transfusions were required after day +11. CD4^+^ T cell reconstitution reached 164 cells/μL on day +63.

The patient’s treatment course was complicated by stomatitis (alleviated by valacyclovir treatment, without recurrence after drug withdrawal on day +68), an episode of uncharacterized low-grade febrile upper airway illness with self-limited azotemia (creatine levels increased to 1.52 mg/dL on day +55, but returned to a baseline level of 0.48 mg/dL 3 days later), and right-side epididymo-orchitis 6 months post-transplant that was resolved via a short course of orally administered cephalosporin. No other significant infections, GVHD, sinusoidal obstruction syndrome, respiratory distress, or other organ dysfunctions were encountered.

**Figure 2:**
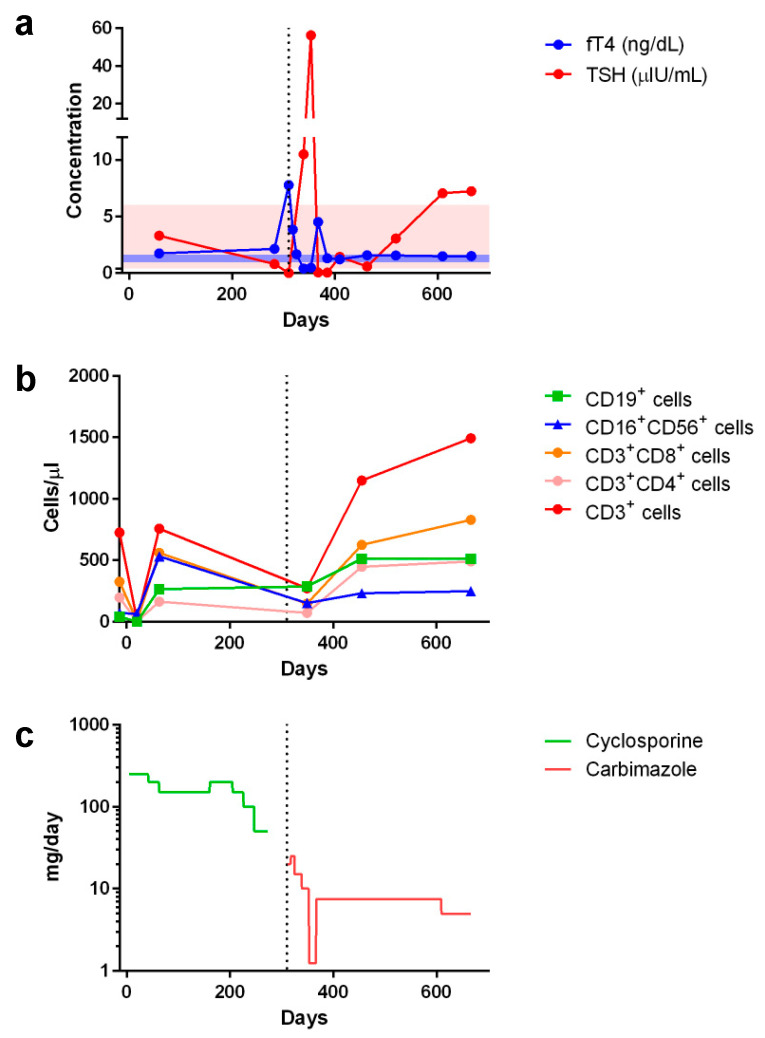
Peri-transplant changes in serum concentrations of (**a**) free thyroxin (fT4) and thyroid stimulating hormone (TSH) as well as (**b**) lymphocyte subset counts in response to (**c**) cyclosporine and carbimazole treatments (showing the comparative dosages). Shaded areas in (**a**) indicate normal ranges of fT4 (blue, 0.925–1.615 ng/dL) and TSH (pink, 0.394–6.00 μIU/mL). Day 0 indicates the day of peripheral blood stem cell infusion. Vertical dotted line indicates when Graves’ disease was diagnosed and respective treatment started.

**Figure 3:**
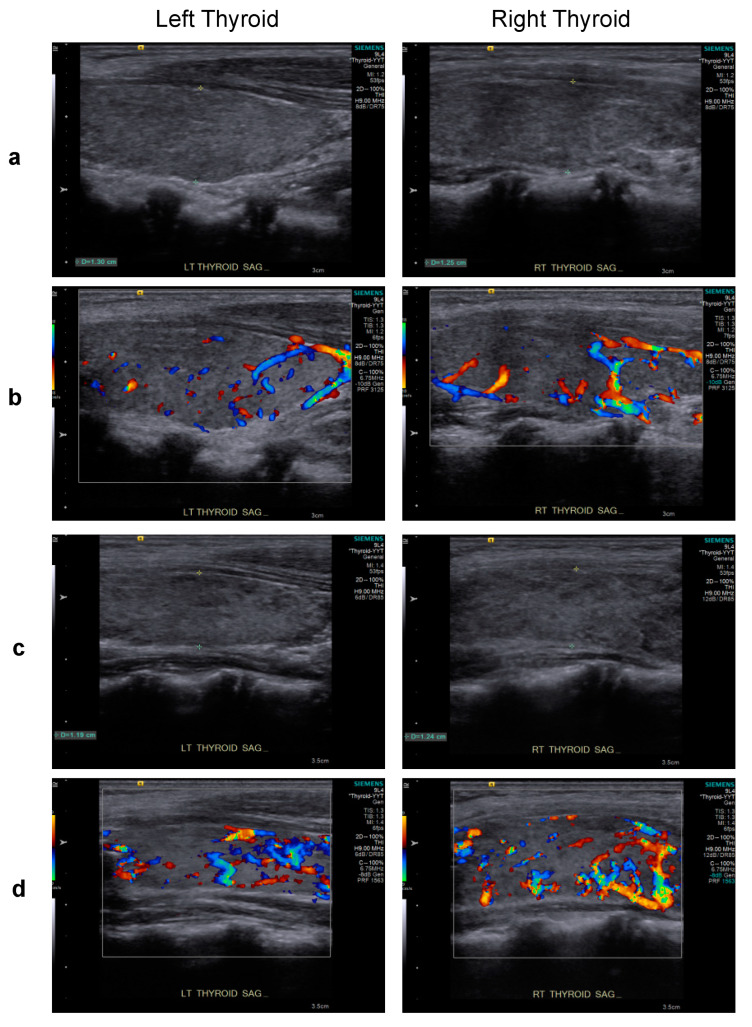
Upon presentation for hyperthyroidism, thyroid sonography of the patient revealed thyroid dimensions at the upper limit of the normal range and mildly heterogeneous parenchymal echotexture (grayscale sagittal images) (**a**) and hyperemia throughout the gland (color Doppler images) (**b**), features characteristic of Graves’ disease. A similar pattern of heterogeneous parenchymal echotexture (**c**) and hyperemia (**d**) were noted after 5 months of carbimazole treatment.

However, Graves’ disease arose dramatically 10 months post-transplant, corresponding to 1 month after tapering and discontinuation of cyclosporine treatment (Figure 2). Graves’ disease was diagnosed when the boy presented with restlessness, rapid speech, poor sleep, orbital swelling, and persistent tachycardia (resting pulse of up to 130 beats/min compared to a baseline of 90–100). An investigation of thyroid function revealed markedly elevated free thyroxin (fT4; >7.77 ng/dL, normal range 0.8–2.3) and total triiodothyronine (T3; 488.90 ng/dL, normal range 90–240), but undetectable thyroid-stimulating hormone (TSH; <0.005 μIU/L, normal range 0.7–6.4). Ultrasonography of the thyroid showed heterogeneous parenchymal echotexture with hyperemia throughout the gland (Figure 3), compatible with Graves’ disease. The patient also displayed markedly elevated levels of antibodies to thyroglobulin (479.0 IU/mL, normal < 115), thyroid peroxidase (293.3 IU/mL, normal < 5.61), and thyrotropin receptors (64.0%, normal 0.0–14.0%), all compatible with the diagnosis of Graves’ disease. The patient’s post-transplant course of immune reconstitution was also affected due to downregulation of lymphocyte subsets soon after Graves’ disease diagnosis, which was more prominent for non-B cell lineages (Figure 2). Immunoglobulin levels remained within normal ranges throughout the post-transplant period (data not shown).

In investigating the patient’s genetic predisposition to Graves’ disease, we identified a *FLT3* c.2500C>T; p.(Arg834Ter) mutation in both the patient and donor (Figure 1a–c). This mutation occurs at a rare population frequency (Figure 1b) and is predicted to elicit premature termination in tyrosine domain 2, indicating that it may result in a truncated protein. Since the *FLT3* stop mutation rs76428106-C, which also results in a truncated protein, has been linked to autoimmune thyroid disease [12], in April 2022, we conducted relevant tests for the donor, which revealed normal telomere length (Figure 1d), fT4 of 1.68 ng/dL, T3 of 121.20 ng/dL, TSH of 1.740 μIU/L, nondetectable antinuclear antibody, normal levels of antibodies to thyroglobulin (19.7 IU/mL), and thyrotropin receptors (6.0%), but borderline elevated antithyroid peroxidase antibodies (7.8 IU/mL). Susceptibility to Graves’ disease among different ethnic groups has been associated with increased frequencies of specific HLA haplotypes [13,14,15,16,17,18,19,20,21], and we identified the donor as carrying HLA-B*46:01 and HLA-DRB1*09:01 and the patient as carrying HLA-C*07 (Table 1). Neither the patient nor the donor hosted HLA haplotypes reported to be protective against Graves’ disease, such as HLA-DRB1*07:01 and HLA-DQA1*02:01 (DR7) (data not shown) [18,19].

Initiating and titrating carbimazole (antithyroid) therapy was begun immediately upon detecting hyperthyroidism, but the trialed rapid tapering failed, as shown in Figure 2, and the boy now requires an oral dosage of 2.5 mg carbimazole twice daily to maintain his euthyroid status. Moreover, follow-up thyroid ultrasonography at 5 months (Figure 3) and 1 year (data not shown) of carbimazole treatment revealed characteristics similar to those at the time of Graves’ disease diagnosis. Follow-up at 6 and 12 months of carbimazole treatment, respectively, indicated that levels of antithyroglobulin antibodies (1062.0 and >4000 IU/mL), thyroid peroxidase (214.6 and 632.1 IU/mL), and thyrotropin receptors (54.0% and 24.0%) remained high compared to those measured when Graves’ disease was diagnosed. No proptosis, periorbital edema, scleral injection, or lid retraction was observed, evidencing inactive or mild orbitopathy. Furthermore, there were no indications of dermatopathy or acropathy. Absolute numbers of lymphocyte subsets recovered during carbimazole treatment (Figure 2), indicating a revival of immune reconstitution. The patient’s telomere lengths improved 1.5 years post-transplant compared to those of a pre-transplant sample (Figure 1d). The boy has returned to school and has remained transfusion-independent since August 2020, with hematological assessment in 2022 revealing a neutrophil count of 2.07–2.95 × 10^9^/L, hemoglobin of 13.0–13.8 mg/dL, and a platelet count of 204–209 × 10^9^/L.

## 3. Discussion

Here, we report the case of a young boy diagnosed with aplastic anemia who developed Graves’ disease after rATG-containing immunosuppressive treatments and haploidentical HSCT from his father. Co-occurrence of aplastic anemia and Graves’ disease, both rare disorders that display similar autoimmune pathogeneses, has been reported previously on several occasions, albeit under different circumstances. For instance, aplastic anemia can arise after Graves’ disease and with or without administration of antithyroid medication [22,23]. Alternatively, Graves’ disease may develop after aplastic anemia, with different mechanisms having been proposed [24,25,26,27]. For example, rATG, which has proven effective in treating steroid-resistant Graves’ orbitopathy [28], has been implicated in promoting the development of Graves’ disease following treatment for aplastic anemia with and without HSCT [24,26]. Adoptive transfer of a pathogenic clone from a donor suffering Graves’ disease [6,7,8], as well as the process associated with GVHD [9], have been postulated as plausible mechanisms by which Graves’ disease evolves upon allogeneic HSCT for aplastic anemia. Nevertheless, neither of those proposed mechanisms seem applicable in the case reported herein; although the donor did display a low titer of antithyroid peroxidase, he exhibited a euthyroid state and an absence of other tested autoantibodies. Indeed, Graves’ disease may evolve 10 months to 8 years following HSCT through variable conditioning regimens, stem cell sources, treatment types, and with or without GVHD (Table 2). We believe that HSCT platforms must lower the threshold level of stimulating anti-thyroid autoimmunity, meaning that, in association with other predisposing factors, Graves’ disease evolves more frequently during immune reconstitution. Interestingly, in our patient’s case, reconstitution of lymphocyte subsets was transiently downregulated as Graves’ disease evolved, as shown in Figure 2.

The pathogenesis of post-HSCT autoimmunity is believed to be multifactorial, involving genetic, infectious, hormonal, and other environmental factors [29]. Many variants of genes involved in immune responses (e.g., HLA, *PTPN22*, *CTLA4*, and *IL2RA*), thyroid function (e.g., *TSHR*, *FOXE1*), and other processes (e.g., *LPP*, *TRIB2*) have been linked to autoimmune thyroid dysfunctions [3]. A large single-center study focused on post-HSCT thyroid autoimmunity, including Graves’ disease and Hashimoto thyroiditis, reported a 2.9% 5-year actuarial rate for autoimmune thyroid dysfunction post-allogeneic HSCT [20]. Thus, investigation of the genetic factors potentially contributing to the development of Graves’ disease in our patient was warranted. We identified germline variants of *FLT3* (carried by both the patient and his HSCT donor) and *SAMD9L* (carried solely by the patient), as well as HLA-B*46:01 and HLA-DRB1*09:01 (carried by the donor) and HLA-C*07 (carried by the patient) haplotypes, together potentially representing genetic predispositions for the evolution of Graves’ disease post-HSCT treatment for aplastic anemia.

*FLT3* encodes fms-related tyrosine kinase 3, a receptor that regulates differentiation and proliferation of hematopoietic progenitor and dendritic cells [30,31]. Notably, mutation at FLT3 residue D835 has been linked to an increased risk of developing acute myeloid leukemia [32,33]. Both our patient and his father carried a germline *FLT3* p.R834* variant, which introduces a premature stop mutation at tyrosine kinase domain 2 and thus represents a loss-of-function germline mutation. Interestingly, the germline intronic rs76428106-C variant of *FLT3*, also representing a loss-of-function mutation, engenders the greatest risk of autoimmune thyroid disease [12]. It has been proposed that this latter intronic variant introduces a novel splice site to curtail FLT3 protein length, resulting in a compensatory increase in levels of its ligand so that it also behaves like a gain-of-function mutation [12]. It would be illuminating to establish if the germline *FLT3* p.R834* variant identified in our patient induces similar effects.

Specific HLA alleles have been associated with incidences of Graves’ disease in different ethnic populations [13,14,15,16,17,18,19,20,21]. In Table 1, we summarize HLA haplotypes reportedly susceptible to Graves’ disease in various ethnic groups. Our HSCT donor possesses HLA-B*46:01 and HLA-DRB1*09:01 haplotypes, both of which have been linked to de novo Graves’ disease and/or to post-HSCT autoimmune thyroid disease in the ethnic Chinese Han population [13,14,20]. Our patient also carries the HLA-C*07 haplotype, although Graves’ disease susceptibility associated with this haplotype has only been documented previously for Caucasians [18,19].

Consistent with reported pediatric myelodysplastic and bone marrow failure syndromes [34,35], our patient also carried a germline *SAMD9L* p.C1267F mutation, which is ultra-rare in terms of population allele frequency (Figure 1b). Gain-of-function *SAMD9L* mutations have been shown to impair multiple pathways and result in enhanced proliferative inhibition and repression of protein translation elongation in primary hematopoietic cells [35,36]. HSCT treatment, either from unrelated cord blood or matched sibling/unrelated bone marrow, has been reported as successful for four of six cases of pediatric myelodysplastic syndromes with germline *SAMD9L* mutations, including one with reduced intensity conditioning [37]. In our patient, bone marrow failure was successfully corrected by nonmyeloablative haploidentical PBSC transplantation from his father, who does not have the *SAMD9L* p.C1267F variant. It remains to be determined how interactions between the host cells carrying *SAMD9L* p.C1267F/*FLT3* p.R834* mutations and the donor cells carrying the *FLT3* p.R834* mutation resulted in Graves’s disease evolving post-HSCT. The donor, who carries both *FLT3* p.R834* mutation and HLA-B*46:01/HLA-DRB1*09:01 haplotypes, has not shown any manifestation of Graves’s disease to date.

In conclusion, the interplay between genetic (the *FLT3* variant carried by the patient and the *FLT3*/HLA-B*46:01/HLA-DRB1*09:01 variants carried by his PBSC donor) and environmental factors arising from the process of immune reconstitution post-haploidentical PBSC transplantation may prompt loss of immune tolerance to thyroid autoantigens, which may have resulted in the development of Graves’ disease in our patient.

## 4. Materials and Methods

### 4.1. Identification of Variants by Whole-Exome Sequencing (WES)

Informed consent was explained and obtained from both the patient and his parents prior to this study. Peripheral blood from the patient and his parents was collected before PBSC transplantation of the patient. One additional peripheral blood sample was obtained from the patient 1.5 years after PBSC transplantation. Genomic DNA from whole blood was isolated using a Wizard genomic DNA purification kit (Promega, Madison, WI, USA) for WES. A DNA library was prepared using a Roche KAPA HyperExome kit (Roche, Basel, Switzerland), and sequencing was performed using a NovaSeq 6000 system (Illumina, San Diego, CA, USA). The sequencing data were aligned to the GRCh37 (hg19) reference genome to identify genetic variants. Mutations were verified by Sanger sequencing. A fragment of the *SAMD9L* gene containing the *SAMD9L* c.3800G>T mutation was amplified by PCR using 5′-TCTCCTAGAAGCTGCGGAAA-3′ and 5′-TGCTGCAGTAGGAAGGCATA-3′ primers, with the former also used for Sanger sequencing. A fragment of the *FLT3* gene containing the *FLT3* c.2500C>T mutation was amplified by PCR using 5′-CACAAAGAACTGCAGCCACC-3′ and 5′-GCCCAAGGACAGATGTGATG-3′ primers. The DNA fragment of the correct size (559 bp) was isolated from the agarose gel for Sanger sequencing using the 5′-CACAAAGAACTGCAGCCACC-3′ primer.

### 4.2. Determination of HLA Haplotypes

Clinical HLA types including low-resolution 2-digit (the donor) and high-resolution 4-digit (the patient) were obtained by Micro SSP™ HLA DNA Typing Trays from LinkSeq^TM^ HLA-ABCDRDQB1 kit with Applied Biosystems^TM^ Standard Block (One Lambda, Inc. West Hills, CA, USA). Next-generation sequencing-based HLA genotypes were extracted from donor and patient WES data using the HLAscan application [38]. In brief, WES sequence reads were aligned with human genome reference hg19 (UCSC browser). Each read in aligned results was classified into a different HLA gene classes via HLAscan application using exon sequence from ImMunoGeneTics project (IMGT)/HLA database (https://www.ebi.ac.uk/ipd/imgt/hla/ (accessed on 4 December 2019)). Reads were aligned with exons 2, 3, 4, and 5 of HLA class I genes, and exons 2, 3, and 4 of HLA class II genes. Allele types were then determined based on the numbers and distribution patterns of the reads for each reference target.

### 4.3. Terminal Restriction Fragment Assay

Leukocyte telomere length was analyzed using terminal restriction fragment assay, as described previously [39]. In brief, leukocyte genomic DNA was digested by *Rsa I* and *Hinf I* restriction enzymes and then resolved by pulsed-field gel electrophoresis. Telomeric DNA was detected by in-gel hybridization using a [^32^P]-labeled telomeric probe.

## Figures and Tables

**Table 1:** HLA haplotypes of the patient and donor (his father) compared to those reportedly susceptible to Graves’ disease.

Susceptible HLA Haplotypes	Ethnicity [References]	Corresponding HLA Haplotypes of Recipient & Donor	Carrier Status of Susceptible HLA Haplotypes in Recipient and Donor
HLA-DQA1 Arg-52	Caucasian [16]	HLA-DQA1 Phe-52 & HLA-DQA1 Phe-52	No and No
HLA-DRB1 Arg-74	Caucasian [17]	HLA-DRB1 Gly-74 & N.D.^1^	No and N.D. ^1^
DRB1*03:01-DQA1*05-DQB1*02 (DR3 haplotype) DRB1*04-DQA1*03-DQB1*03 (DR4 haplotype)	Caucasian [15,21] Caucasian [15]	DRB1*11:01:01/11:01:01-DQA1*05:09/05:09-DQB1*03:01:01:03/03:01:01:03 & DRB1*11:01:01/09:01:02-DQA1*05:05:01:02/03:02-DQB1*03:01:01:01/03:03:02:04	No and No No and No
DPB1*05:01	Chinese Han [13]	DPB1*02:02/02:01:02 & DPB1*02:02/02:01:02	No and No
DQB1*05:02 DQB1*02	Chinese Han [13] Caucasian [15]	DQB1*03:01:01:03/03:01:01:03 & DQB1*03:01:01:01/03:03:02:04	No and No No and No
DRB1*15:01 DRB1*16:02 DRB1*09	Chinese Han [13] Chinese Han [13] Chinese Han [20]	DRB1*11:01:01/11:01:01 & DRB1*11:01:01/09:01:02	No and No No and No No and Yes
B*05 B*46:01 B*08	Chinese Han [14] Chinese Han [13,14] Caucasian [15]	B*39:01:01:01/51:02:01 & B*46:01:01/51:02:01	No and No No and Yes No and No
C*07 (Cw7)	Caucasian [15]	C*07:02:01:03/15:02:01:02 & C*01:02:30/15:02:01:03	Yes and No

N.D., not determined; ^1^ no read in that region from the donor’s whole-exome sequencing data.

**Table 2:** Characteristics of patients who have developed Graves’ disease following allogeneic HSCT for severe aplastic anemia.

Age (Years) at HSCT/Sex	Disease at HSCT	HSCT Donor/Source	HSCT Regimens *	HSCT-GD Interval	GD Risk Factors	GD Outcome	Reference
8.5/M	SAA refractory	Haplo/PBSC	Flu150/Cy29/TBI4—PTCy/CSA/MMF	10 months	Described in text	Euthyroid over 1 year under carbimazole	Present case
4/M	SAA at diagnosis	MFD/BMSC	Cy200/rATG—N.A.	3 years	rATG, DR9	Under methimazole	[11]
8/F	SAA at diagnosis	MSD/BMSC	Cy200/rATG—CSA/MTX	3 years	rATG	Euthyroid over 1.5 years after methimazole/thyroidectomy and under L-thyroxine	[10]
14/M	SAA at diagnosis	MSD/BMSC	Cy200/rATG—CSA/MTX	2 years	rATG, donor GD, HLA-DRB1*0301 (DR3)	Euthyroid over 2 years after lithium carbonicum/thiamazole/thyroidectomy	[6]
17/F	SAA at diagnosis	MSD/BMSC	Cy200/TLI7—CSA	30 months	Donor GD, HLA-Bw46	Clinical improvement but persistent T3 elevation over one year after thiamazole	[8]
10/M	SAA at diagnosis	MSD/BMSC	Cy—MTX	8 years	Donor GD	N.A.	[7]
50/M	SAA at diagnosis	MSD/BMSC	Cy180—MTX	36 months	GVHD	Euthyroid after propranolol/I^131^ 15 mCi and under thyroxine	[9]

BMSC, bone marrow stem cells; CSA, cyclosporine; Cy, cyclophosphamide; F, female; Flu, fludarabine; GD, Graves’ disease; GVHD, graft-versus-host disease; Haplo, HLA haploidentical; HLA, human leukocyte antigen; HSCT, hematopoietic stem cell transplantation; M, male; MFD, HLA-matched family donor; MSD, HLA-matched sibling donor; MTX, methotrexate; N.A., not available; PBSC, peripheral blood stem cells; rATG, rabbit anti-thymocyte globulin; SAA, severe aplastic anemia; TBI, total body irradiation; TLI, total lymphoid irradiation. * HSCT regimen includes conditioning—GVHD prophylaxis. Numbers following Cy, Flu, or TBI/TLI denote total mg per kg body weight given, total mg per m^2^ body surface area given, and TBI/TLI, total irradiation given, respectively.

## Supplementary Materials

The following supporting information can be downloaded at: https://www.mdpi.com/article/10.3390/ijms23169494/s1.
