# Supplementary material for: Evolution of Graves’ Disease during Immune Reconstitution following Nonmyeloablative Haploidentical Peripheral Blood Stem Cell Transplantation in a Boy Carrying Germline SAMD9L and FLT3 Variants
Source: Int J Mol Sci. 2022 Aug 22;23(16):9494. doi: 10.3390/ijms23169494 (PMC9409095; doi:10.3390/ijms23169494)
Supplement: Supplementary file 1 [file ijms-23-09494-s001.zip › ijms-1853556-supplementary.pdf]

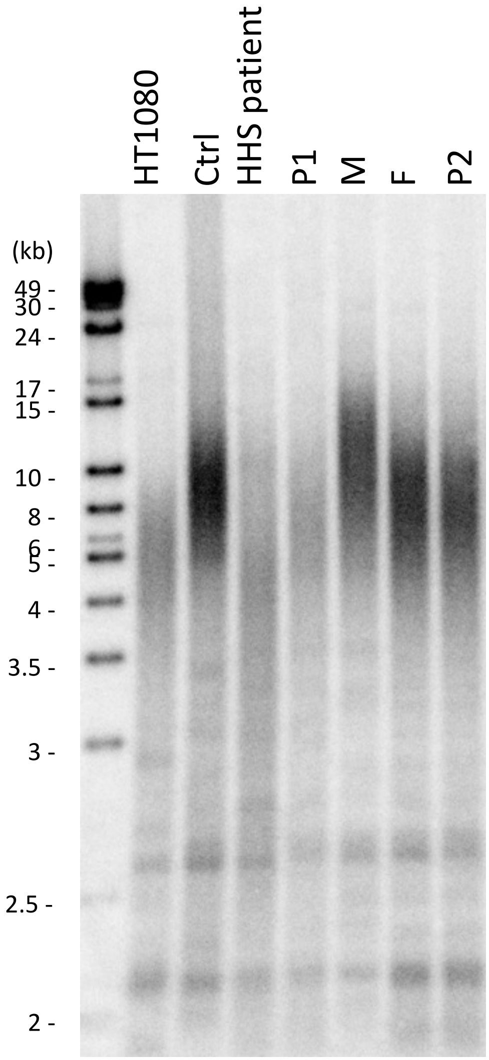

Figure S1. Telomere length analysis (by terminal restriction fragment assay) of DNA from leukocytes of the patient pre-transplant (P1) and 1.5 years post-transplant (P2), patient's father (F), patient's mother (M), an age-matched healthy control (Ctrl), an age-matched patient with Hoyeraal-Hreidarsson syndrome (HHS patient) and from HT1080 fibrosarcoma cancer cells.
